# Trends in Adolescent Health Risk Behaviors and Wellbeing: A 10 Year Observation from the EDIT Surveillance of Tuscany Region, Italy

**DOI:** 10.3390/ijerph19116863

**Published:** 2022-06-03

**Authors:** Vieri Lastrucci, Marco Lazzeretti, Francesco Innocenti, Chiara Lorini, Alice Berti, Caterina Silvestri, Fabrizio Chiesi, Annamaria Schirripa, Sonia Paoli, Giulia Di Pisa, Andrea Moscadelli, Guglielmo Bonaccorsi, Fabio Voller

**Affiliations:** 1 Epidemiology Unit, Meyer Children’s Hospital, Viale Gaetano Pieraccini 24, 50139 Florence, Italy; vieri.lastrucci@gmail.com; 2 Department of Health Sciences, University of Florence, Viale GB Morgagni 48, 50134 Florence, Italy; chiara.lorini@unifi.it; 3 Epidemiologic Observatory, Regional Health Agency of Tuscany, Via Pietro Dazzi 1, 50141 Florence, Italy; marco.lazzeretti4@virgilio.it (M.L.); francesco.innocenti@ars.toscana.it (F.I.); alice.berti@ars.toscana.it (A.B.); caterina.silvestri@ars.toscana.it (C.S.); fabio.voller@ars.toscana.it (F.V.); 4 Medical Specialization School of Hygiene and Preventive Medicine, University of Florence, 50134 Florence, Italy; fabrizio.chiesi@unifi.it (F.C.); annamaria.schirripa@unifi.it (A.S.); sonia.paoli@unifi.it (S.P.); giulia.dipisa@unifi.it (G.D.P.); andrea.moscadelli@unifi.it (A.M.)

**Keywords:** adolescents, health risk behaviors, surveillance system, substance abuse, sexual behaviors

## Abstract

*Background*: The aim of this study was to evaluate the trends of prevalence of health risk behaviors (HRBs) and health conditions over a 10 year period (2008–2018) in a representative sample of adolescents of Tuscany Region, Italy. *Methods*: This was a repeated cross-sectional (four survey waves) study. The prevalence of 17 HRBs and health conditions were analyzed by age, sex, and socioeconomic status (SES). *Results*: A total of 21,943 students were surveyed. During the study period, decreases in smoking participation, cocaine use, driving under the influence of alcohol and drugs, and problem gambling were observed, while alcohol abuse and at-risk sexual behaviors remained unchanged or increased. Males resulted more frequently involved in most of the HRBs, while females more frequently reported physical inactivity, regular smoking, and not using a condom. Female participation in smoking and alcohol abuse behaviors, fruit and vegetable consumption, and bullying worsened over the study period. Smoking, poor dietary habits, physical inactivity, high distress level, and obesity were more frequently observed in low-SES students than in high-SES students. *Conclusions*: The findings showed different tendencies in adolescent participation in HRBs over the last decade; concerning trends in at-risk sexual behaviors and alcohol consumption and females’ risk-taking behavior on the rise require careful monitoring.

## 1. Introduction

Lifestyle habits and health risk behaviors (HRBs) contribute to substantial health problems for adolescents and impact their health in subsequent phases of life, as they often persist into adulthood [1]. Consequences of HRBs extend beyond health as they can influence school performance and, therefore, occupational and financial prospects, which are in turn interrelated with health in adulthood [2,3,4]. Therefore, investing in the promotion of healthy lifestyles and behaviors in adolescent populations is a crucial strategy for public health and society at large.

HRBs in adolescents can be grouped in different categories associated with leading causes of morbidity and mortality in adolescence and later in adulthood. In particular, the US Centers for Disease Control and Prevention (CDC) groups HRBs in the following six categories: behaviors that contribute to unintentional injuries and violence; tobacco use; alcohol and other drug use; sexual behaviors that related to unintended pregnancy and sexually transmitted infections; unhealthy dietary behaviors; physical inactivity [5]. Various factors are reported to influence the adolescent participation in HRBs; in particular, age and socioeconomic status are commonly reported among the main determinants of HRBs participation in adolescents [6,7]. Furthermore, HRB participation and wellbeing in adolescents seem to be highly patterned by sex, with males showing a higher level of participation in substance use and behaviors that may contribute to unintentional injuries and violence, and females showing a poorer level of physical activity and mental health [8,9,10,11].

Surveillance systems periodically collecting population-based data on HRBs are, thus, essential to evaluate HRBs and their determinants in adolescents, to identify at-risk groups, and to establish priorities in health interventions and policies. Furthermore, the results arising from surveillance over time provides key information on changes and trends of HRBs in selected populations and allow policymakers and public health professionals to evaluate the effectiveness of health interventions and policies. For some HRBs, results from surveillance systems from different countries seem to show common tendencies in their participation over time; indeed, declining trends are emerging in tobacco use and consumption of illicit drugs (other than cannabis), while it appears that little progress has been made in terms of participation in physical activity, which has remained particularly low over the time; moreover, condom use and a variety of indicators on mental health issues are showing worrying trends [12,13,14,15,16]. However, for most of the HRBs, such clear common trends are not traceable; this is especially true for age, sex, and socioeconomic inequalities in HRB participation, probably reflecting differences in health policies and interventions, as well as other influencing sociocultural conditions across regions and countries [5,12,13].

Italian adolescents have a concerningly low participation level in physical activity, and more than half of them eat neither fruits nor vegetables daily [17]; furthermore, a considerable proportion of adolescents reported a high frequency of participation in various unsafe and risky driving practices [18]. Italian adolescents are reported to have one of the highest participation levels in tobacco and cannabis consumption across the countries surveyed by the Health Behavior in School-Aged Children (HBSC) survey in 2017/2018; in the HBSC survey, several indicators also showed that Italian adolescents are experiencing a poor mental health level [17].

Various surveillance systems are currently monitoring HRBs in adolescents across the world [5,12,13]; in Tuscany Region, Italy, the Regional Health Agency (ARS Toscana) has developed a surveillance system called EDIT. EDIT surveillance is officially recognized by the Italian Ministry of Health and is aimed at monitoring the HRBs and health conditions of Tuscan adolescents. The aim of the present study was to describe the results from the survey waves carried out over the last decade. In particular, the study aimed to describe the trends of prevalence of several HRBs and health conditions, and to evaluate age, sex, and socioeconomic status trend changes over a 10 year period in a representative sample of adolescents of Tuscany Region, Italy.

## 2. Materials and Methods

The study is based on data derived from the EDIT (Epidemiologia dei Determinanti dell’Infortunistica Stradale Toscana—epidemiology of the determinants of road traffic accidents in Tuscany Region) surveillance system. EDIT surveillance was approved for research purposes by the Decree of the President of the Council of Ministers of Italy (Decreto del Presidente del Consiglio dei Ministri—DPCM) on 3 March 2017. The study was conducted according to the principles described in the Declaration of Helsinki.

### 2.1. Study Population

This was a cross-sectional study based on the data collected in four different waves of the EDIT survey (i.e., 2008, 2011, 2015, and 2018; each wave was carried out from February to May of the same year). The EDIT surveillance survey investigates the epidemiology of several health and risk-taking behaviors and of their determinants in a representative sample of students attending the upper secondary schools of the Tuscany Region, Italy. The EDIT surveillance adopts a stratified sampling method according to the administrative areas of the Tuscan Health System and the type of secondary school. Further details concerning the sampling methodology are described elsewhere [8]. In the last survey wave, a total of 6824 students participated, representing 3.55% of the population aged 14–19 in the Tuscany Region. The response rate was above 96% in all considered survey waves.

### 2.2. Data Collection and Measurements

Students who gave their consent were asked to fill an anonymous self-administered questionnaire during schooltime. The questionnaires had the same structure in the different survey waves, with only a few questions varying across waves. In particular, the questionnaires ranged from 71 to 82 questions across the survey waves considered (average completion time of 45 min). The questionnaire was self-administered in the classroom by means of special tablets (tablet-assisted self-interview—TASI) using wireless technology. In each survey wave, the questionnaire presented 11 sections covering the following topics: sociodemographic information, social wellbeing and mental health, driving behaviors and road traffic accidents, health behaviors (smoking, alcohol consumption, recreational drug consumptions, physical activity, and dietary habits), and risk-taking behaviors (gambling, bullying, sexual behaviors, and risky riding behaviors).

Sociodemographic variables included sex, age, educational level of the parents (preschool education, primary education, or lower secondary education; upper secondary education; bachelor’s degree, master’s degree’ or higher). For this study, the highest education level of both parents was considered as a measure of the family socioeconomic status (SES) and coded as follows: low (preschool education, primary education, and lower secondary education), medium (upper secondary education), and high (bachelor’s degree, master’s degree, or higher). Mental health was investigated using the six-item Kessler Pyschological Distress Scale (K-6) [19]; this tool allows evaluating a self-report measure of psychological distress (dichotomized: a score < 19 indicates no or low psychological distress; a score ≥ 19 indicates high psychological distress).

Health behavior variables included smoking (regular smoker, occasional/social smoker, nonsmoker), dietary habits assessed by consumption of fruits (at least once a day, a few times a week, hardly ever or never) and vegetables (at least once a day, a few times a week, hardly ever or never), being drunk in the last year (no, yes), binge drinking in the last month (defined as the consumption of five or more drinks in a row on an occasion; response options: no, yes), use of cannabis or cocaine in the last month (no, yes), physical activity (defined as any bodily movement produced by skeletal muscles that requires energy expenditure; response options: less than once a week, more than once a week). As far as risk-taking behaviors were concerned, variables included being a bully and being bullied in the last year (no, yes), sexual intercourse initiation before age 14 (no, yes), use of condom during the last sexual intercourse (no, yes), and driving under the influence (DUI) of alcohol or drugs in the last year (no, yes). Furthermore, gambling behavior was assessed using the Lie/Bet Screening Instrument [20], which identifies participants at risk of pathological gambling behaviors. Lastly, self-reported height and weight were determined in order to calculate the body mass index (BMI) using the following formula: weight (kg)/height × height (m^2^).

### 2.3. Statistical Analysis

Data were weighted by gender, age, and area (ASL or Health District according to the survey edition) to infer data with respect to a representative sample of the population aged 14–19 in Tuscany Region. Responses to the questionnaire were evaluated across the different survey waves to assess changes in behaviors of Tuscan students. In particular, for each variable, data were reported as the percentage and 95% CI. Associations between the edition’s year and the variables were investigated by chi-square’s test for weighted data (Pearson chi-square (χ^2^) statistic corrected for the survey design with the second-order correction of Rao and Scott and converted into a F statistic). First, the analysis was performed for the total sample, and then by sex, high-school years (the lower two grade levels “biennium” and the three higher grade levels “triennium”), and SES level. For each analysis, an α level of 0.05 was considered as significant. The statistical software STATA (StataCorp. 2017 Stata Statistical Software: Release 15. College Station, TX, USA: StataCorp LLC) was used for data analyses.

## 3. Results

A total of 21,943 students were tested from 2008 to 2018 (51.7% male and 48.3% female) (Table 1).

Table 2 reports the prevalence of health-related behaviors in the four survey waves considered. In some of the explored areas, it is possible to observe that trends over time are clearly decreasing; these include declines in the prevalence of students who are regular smokers (21.9%, 95% CI 20.6–23.3 in 2008; 19.2%, 95% CI 18.2–20.3 in 2018), had used cocaine in the last month (3.0%, 95% CI 2.6–3.5 in 2008; 1.5%, 95% CI 1.2–1.8 in 2018), and had a case of DUI of alcohol or drugs during the last year (DUI of alcohol: 20.5%, 95% CI 19.2–21.9 in 2008; 5.5%, 95% CI 5.0–6.2 in 2018. DUI of drugs: 11.5%, 95% CI 10.5–12.6 in 2008; 5.1%, 95% CI 4.6–5.7 in 2018). Furthermore, the results from Lie/Bet scores showed a decreasing trend in the percentage of students at risk of pathological gambling in the last decade (10.5%, 95% CI 9.6%–11.6% in 2008; 6.8%, 95% CI 6.2–7.5 in 2018). On the other hand, for some behaviors, it was possible to observe a flat or increasing trend in the percentages of students reporting their participation; these include binge drinking (32.3%, 95% CI 30.7–33.8% in 2008; 33.4%, 95% CI 32.1–34.7 in 2018), being drunk (46.7%, 95% CI 45.1–48.4 in 2008, 48.2%,95% CI 46.8–49.6), and not using a condom during the last sexual intercourse (32.3%, 95% CI 30.1–34.5 in 2008; 41.6%, 95% CI 39.5–43.8 in 2018). Furthermore, compared with 2008 survey results, the percentage of students reporting being bullied was slightly higher in 2018 (20.6%, 95% CI 19.3–22.1; 23.0, 95% CI 21.8–24.2), while the percentage of students reporting being a bully was relevantly lower in 2018 (18.7%, 95% CI 17.4–20.1 in 2008; 11.9%, 95% CI 11.0–12.8 in 2018).

The prevalence of health-related behaviors by sex in the four survey waves is reported in Table 3. In the most recent survey wave, males reported a significantly higher participation in bullying behavior, binge drinking, cannabis use, physical activity, DUI of alcohol, and DUI of drugs compared to females; furthermore, a higher percentage of males were at risk of pathological gambling (Table 3). On the other hand, a higher percentage of females reported regularly smoking, not having used a condom during the last sexual intercourse, eating vegetables and fruits daily, and being bullied (Table 3). Over the course of the study period, the following behaviors and conditions showed prevalence trends with opposite directions between males and females: excessive alcohol consumption-related behaviors (binge drinking and being drunk), physical activity, and being bullied. The prevalence of all these behaviors and conditions increased in females and declined in males over the course of the study period. Furthermore, although the participation in regular smoking, at-risk pathological gambling, bulling behaviors, and DUI of alcohol or drugs showed declining trends in both sexes during the observed period, these declines were sharper in males than in females.

Table 4 reports the prevalence of health-related behaviors by high-school years. Students enrolled in the triennium years showed a significantly higher participation in all the considered risky behaviors, with the exception of bullying behaviors. Furthermore, students in the biennium reported a higher participation in physical activity, and no significant differences were observed in the daily consumption of fruits and vegetables between the biennium and triennium students. Cannabis consumptions increased significantly over the study period in the biennium students (10.8%, 95% CI 9.3–12.5 in 2008; 16.2%, 95% CI 14.5–18.0 in 2018), while it remained stable in the triennium students (34.6%, 95% CI 32.9–36.3 in 2008; 34.4%, 95% CI 32.8–35.9 in 2018).

Table 5 reports the prevalence of health-related behaviors by SES. In 2018, regular smokers in the low-SES category were higher than in the medium- and high-SES categories. Over the course of the study period, the prevalence of regular smoking in low-SES students remained stable, while a declining prevalence trend was observed for regular smoking in high-SES students. Low-SES students reported being significantly less frequently involved in physical activity and fruit and vegetable consumption compared with high-SES students in 2018. Trends for vegetable consumption showed that the proportion of students reporting a daily consumption declined in low-SES students, while it remained stable in high-SES students. As for the other HRBs considered, participations by SES showed similar trends over the study period.

As for the health conditions explored, the prevalence of obesity and overweight was 2.3% (95% CI 2.0–2.8) and 12.7% (95% CI 11.8–13.6) in 2018, respectively (Table 2); these percentages were similar to those registered in the previous survey waves. Sex differences in the proportion of underweight and overweight were observed (Table 3), with females showing a higher proportion of underweight (females: 4.0% 95% CI 3.3–4.9; males: 1.7%, 95% CI 1.3–2.3) and males showing a higher proportion of overweight (males: 16.2%, 95% CI 14.8–17.6). Furthermore, the prevalence of obesity was higher in low-SES students compared with high-SES students in all survey waves (Table 5). Over the course of the study period, it was possible to observe an increase of the percentage of students reporting a high distress level (Table 2), from 18.5% (95% CI 17.4–19.7) in 2008 to 21.7% (95% CI 20.6–22.9) in 2018. In the most recent survey wave, higher proportions of a high level of distress were reported in female students (32.7%, 95% CI 30.8–34.6), students enrolled in the triennium (23.5%, 95% CI 22.1–24.9), and low-SES students (25.6%, 95% CI 22.9–28.5) compared with males (11.5%, 95% CI 10.4–12.7), biennium students (19.1%, 95% CI 17.3–21.1), and high-SES students (19.7%, 95% CI 17.8–21.7), respectively (Table 3, Table 4 and Table 5); furthermore, the proportion of students reporting a high distress level increased relevantly in females and in low-SES students during the study period, while it remained substantially unvaried in males and high-SES students (Table 3 and Table 5).

## 4. Discussion

The aim of the study was to evaluate the trend of prevalence of HRBs and health-related conditions in adolescents over the course of 10 years and to examine age, sex, and socioeconomic status variations. Data from the EDIT surveillance system of Tuscany Region, specifically data from the past four survey waves, were used (2008–2018). For some HRBs, results allowed identifying temporal trends in their overall prevalence, while, for others, no clear trends were observed. In particular, results showed a declining trend of the prevalence of students who regularly smoke, had used cocaine, had a case of DUI of alcohol and drugs, and were at risk of pathological gambling. Secondly, unchanged or increasing prevalence trends were observed for alcohol abuse and at-risk sexual behaviors. As for sex differences in practicing HRBs, during the most recent survey, males resulted more frequently involved in most of the HRBs considered, while females more frequently reported physical inactivity, regular smoking, and not using a condom during the last sexual intercourse. However, for almost all HRBs, the participation gap between males and females was reduced during the study period; smoking behavior was among the exceptions to this trend. The percentage of students declaring regularly smoking declined in males while it remained almost unvaried in females over the study period. As for health-related conditions, a significant increase in the percentage of females who experienced a high level of distress was observed during the study period. As for SES differences in HRB participation, smoking, poor dietary habits, physical inactivity, high distress level, and obesity were more frequently observed in low-SES students than in high-SES students. Lastly, worsening trends in smoking, dietary habits, and distress level were observed in low-SES students.

Results of our study showed that, over the past decade, adolescents have moved toward healthier choices in several areas; this was particularly evident for tobacco use, DUI of alcohol and drugs, and cocaine consumption. For these HRBs, our results confirm those from other surveillance systems, indicating a constant decline in their adoption [12,13,14]. Tobacco control, road traffic accident prevention, and illicit drug consumption are among the top priorities in the public health agenda for youth in Italy and internationally [21,22]; our results confirm a regional and international positive tendency in these fields. Another positive tendency that emerged from our findings is the reduction in the prevalence of problem gambling over the study period. Gambling among adolescent has been an understudied public health issue until recently when concerns related to the development of new forms of gambling via the Internet and their advertisements began to rise [23,24]. The reassuring tendency in problem gambling that emerged in our study may be explained, at least in part, by the strengthening on legislation on youth gambling and by specific prevention program implemented in Italy and in Tuscany over the past decade [25].

On the other hand, our results showed concerning trends for alcohol abuse, risk sexual behaviors, physical activity, and dietary habits. As for alcohol abuse behaviors, stable trends at a rather high level were observed for both binge drinking and drunkenness, with older students and girls resulting more prone to these behaviors. Although alcohol consumption is highly influenced by sociocultural factors, our results on alcohol abuse appear to be in line with data from other countries [12,13], highlighting a global phenomenon. Another increasing tendency, in both females and males, was observed in the percentage of students not using a condom. This confirms findings from other surveillance systems reporting declining trends in condom use in several countries [5,12]. The high cost of condoms, misinformation, social situation, and religious beliefs may influence adolescents’ decisions and contribute to a lower use of contraceptive methods [26]. Furthermore, in line with other studies, females showed less frequent use of condoms than males in our study [12,27]. A possible explanation may be linked to the fact that females are more frequently sexually active with older partners than males [28]. The low but stable level of physical activity, as well as fruit and vegetable consumption, observed in our study confirms that the adoption of poor eating habits is often associated with high levels of sedentary behavior in adolescents [29]. Taken together, these findings highlight that HRBs requiring to be carefully monitored have a variegated nature; thus, health promotion interventions with a multi-risk approach that intervene on strengthening transversal competences and skills, such as health literacy and life skills, in multiple community settings, are likely to be effective in achieving healthier choices in adolescents [30,31,32,33].

Several sex differences were present in the HRB participation during the last survey wave, with males showing higher participation in most of the cases. However, for most of HRBs, this sex gap was narrowed over the past decade. For several HRBs, this trend of reduced sex differences indicates a movement toward healthier choices in which both sexes showed a reduced prevalence of participation over time; however, for some HRBs, the sex gap reduction over time represented a step backward, in which one sex moved toward the worse level of the other sex. This was the case for binge drinking, as well as fruit and vegetable consumption, in which female habits worsened over the time, getting closer to the male levels. Only three HRBs showed an increasing sex gap over the study period—smoking, being drunk, and being bullied; the augmented gaps were due to opposite trends between sexes, with females experiencing a worsening level over the study period, except for smoking, in which females showed a stable level while males declined. Answers about bullying behaviors showed contrasting results; while both sexes stated less frequently being a bully, being bullied was an increasing phenomenon for girls, especially in older classes. Findings from other studies showed that sex difference in bullying behaviors are largely variable and may reflect cultural and social differences among countries [34]. Taken together, these findings on sex differences in HRBs highlight the importance of understanding the context and challenges that each sex experience and the need to incorporate gender specificity in policies and programs addressing HRBs in youth.

Our findings also showed an increasing sex gap in distress levels experienced by adolescents. The percentage of females who experienced a high level of distress was constantly higher than males and increased over the study period. This finding appears similar to other European surveys [35]; females more frequently experience depression and anxiety and feel more socially pressured than males. Distress among adolescents, particularly in females, can also be associated with pathological use of social media and with the recently emerging phenomena of cyberbullying and body shaming online [36]. Stressful conditions can also be a result of a negative school environment; results from a recent World Health Organization survey showed that Italian adolescent students reported one of the worst levels of stress by schoolwork [11].

As for age differences, older adolescents were more frequently involved in HRBs than younger adolescents; this is in line with the literature, except for bullying behaviors in which our study confirmed that bullying tends to peak during middle school years and appears to decrease by the end of high school [37]. Worrying trend emerged in the marijuana consumption in the younger adolescents, while, in older adolescents, this habit remained relatively stable over time. Interestingly, this increase in consumption in younger adolescent started during the period in which the commercialization of low-THC marijuana strains was legalized in Italy (Italian Law n. 242/2016). Although the effect of legalized marijuana on the pattern of adolescent use is still unclear, some authors argued that it may lead to an increased consumption due increased availability, greater social acceptance, and possibly lower prices [38]. Our results lead to a hypothesis that the potential effects of these factors—if present—are stronger in the younger adolescents.

Contradictory findings concerning the relationship between SES and HRB participation in adolescents have been reported, with some studies reporting an inverse relationship and others reporting a higher participation in high-SES adolescents than in other SES groups [7,39,40]. In our study, high distress level and obesity were more frequently observed in low-SES than in high-SES students. The higher level of obesity observed in low-SES students is probably related to the poorer dietary habits and physical activity levels observed in this groups. As for high distress level, worsening trends over the study period were observed in low-SES students; this finding needs to be carefully monitored and further studied not only for the mental health issues but also for the reciprocal relationships with HRB participation; indeed, several studies reported that a high distress level influences participation in HRBs and vice versa, which may create a vicious circle leading to increasing health inequalities [7,41,42].

The present study had several strengths and limitations. As for the strengths, the study assessed the prevalence of a comprehensive range of HRBs in a large and representative sample of adolescents over a 10 year periods. Furthermore, each survey wave had a high participation rate. As far as the limitations are concerned, the surveys were based on self-reported data; therefore, results may have been influenced by recall and social desirability bias of adolescents. However, it should be pointed out that the anonymity and the self-administration of the survey may have limited the impact of social desirability bias. Lastly, the temporal trends described were derived from repeated cross-sectional surveys; therefore, the results are jointly influenced by biological differences among adolescents, shared experiences and historical contexts of cohorts, and factors related to the moment in which the observation occurred; the individual effects of each factor could not be determined.

## 5. Conclusions

Findings of the study showed various different tendencies in adolescent participation in HRBs over the course of the last decade. More specifically, comforting trends toward healthier choices were seen in the overall adolescent participations in several HRBs and in the reduction in sex differences in most of the HRBs. These findings probably highlight that public health efforts addressing HRBs in adolescents were effective, particularly for the most commonly acknowledged HRBs such as tobacco consumption and impaired driving states. However, concerning trends emerged in the overall adolescent participation in at-risk sexual behaviors and alcohol consumption, as well as in marijuana consumption in younger adolescents; furthermore, worrying trends in the prevalence of high distress level and in regular smoking were observed in females and low-SES students. Further studies on these worsening trends are needed to better clarify the possible cultural, socioeconomic, and environmental determinants. Furthermore, these findings highlight the need of health promotion and prevention interventions tailored to specific HRBs and population groups. In particular, integrated programs, which involve schools, families, community, and healthcare settings, and gender-specific interventions are likely to be effective in achieving healthier choices in adolescents.

## Figures and Tables

**Table 1:** Sociodemographic characteristics of the study sample (survey waves 2008, 2011, 2015, and 2018).

Survey Wave	*N*	Males	Age					Socioeconomic Status		
			*14 Years*	*15 Years*	*16 Years*	*17 Years*	*18 Years*	*Low*	*Medium*	*High*
2008	5213	51.6%	19.2%	20.0%	20.0%	20.5%	20.4%	25.8%	47.4%	26.8%
2011	4829	51.6%	19.8%	19.5%	19.7%	19.8%	21.2%	21.9%	46.8%	31.3%
2015	5077	51.7%	20.5%	19.8%	20.0%	19.8%	19.8%	21.2%	47.3%	31.5%
2018	6824	51.8%	20.2%	20.1%	20.3%	19.6%	19.9%	19.9%	48.4%	31.7%
Total	21,943	51.7%	19.9%	19.9%	20.0%	19.9%	20.3%	22.2%	47.4%	30.4%

**Table 2:** Overall trends of the prevalence of health risk behaviors and health conditions (survey waves 2008, 2011, 2015, and 2018).

	2008	2011	2015	2018	*p*
**Lie/Bet**	10.54 (9.6–11.6)	9.69 (8.8–10.6)	6.73 (6.1–7.5)	6.78 (6.2–7.5)	<0.0001
**Smoke**					
Regular smokers	21.9 (20.6–23.3)	21.2 (19.9–22.4)	20.5 (19.3–21.7)	19.2 (18.2–20.3)	<0.01
Occasional smokers	13.9 (12.9–15.0)	13.8 (12.8–14.9)	15.9 (14.8–17.0)	14.9 (13.9–15.9)	
Nonsmokers	64.1 (62.6–65.7)	65 (63.5–66.5)	63.7 (62.2–65.1)	65.9 (64.6–67.2)	
**Binge drinking at least once in the last month**	32.3 (30.7–33.8)	32.4 (30.9–33.9)	31.8 (30.4–33.2)	33.4 (32.1–34.7)	0.45
**Being drunk at least once in the last 12 months**	46.7 (45.1–48.4)	47.8 (46.2–49.4)	47.2 (45.7–48.8)	48.2 (46.8–49.6)	0.58
**Being bullied in the last 12 months**	20.6 (19.3–22.1)	19.6 (18.4–20.9)	20.1 (18.9–21.3)	23 (21.8–24.2)	0.001
**Being a bully in the last 12 months**	18.7 (17.4–20.1)	19.1 (17.9–20.4)	17.1 (16.0–18.3)	11.9 (11.0–12.8)	<0.0001
**BMI categories**					
Obese	2.7 (1.9–3.7)	2.2 (1.8–2.7)	3.2 (2.7–3.8)	2.3 (2.0–2.8)	0.05
Overweight	12.7 (11.5–13.9)	12.6 (11.6–13.7)	12.6 (11.6–13.7)	12.7 (11.8–13.6)	
Normal	81.5 (80.0–83.0)	82.8 (81.6–84.1)	80.6 (79.4–81.8)	82.2 (81.1–83.2)	
Underweight	3.1 (2.6–3.8)	2.4 (1.9–3.0)	3.6 (3.1–4.2)	2.8 (2.4–3.3)	
**High distress level**	18.5 (17.4–19.7)	16.3 (15.2–17.5)	21 (19.8–22.2)	21.7 (20.6–22.9)	<0.0001
**Early sex (before 14 years old)**	3.6 (3.1–4.3)	3.1 (2.6–3.7)	3.3 (2.8–3.9)	3 (2.6–3.5)	0.36
**No condom use during the last sexual intercourse**	32.3 (30.1–34.5)	37.4 (35.1–39.8)	41.2 (38.9–43.5)	41.6 (39.5–43.8)	<0.0001
**Use of cannabis in the last month**	25.3 (24.0–26.6)	21.5 (20.3–22.8)	28.2 (26.9–29.5)	27 (25.9–28.2)	<0.0001
**Use of cocaine in the last month**	3 (2.6–3.5)	2.5 (2.0–3.0)	2 (1.7–2.5)	1.5 (1.2–1.8)	<0.0001
**Physical activity**					
At least once a week	72.3 (70.9–73.7)	74 (72.6–75.3)	75.1 (73.8–76.4)	73.8 (72.5–74.9)	0.03
Less than once a week/hardly ever/never	27.7 (26.3–29.1)	26 (24.7–27.4)	24.9 (23.6–26.2)	26.2 (25.1–27.5)	
**Eating vegetables**					
At least once a day	45.5 (43.9–47.1)	43.2 (41.7–44.8)	40.9 (39.4–42.4)	43.6 (42.3–45.0)	<0.0001
A few times a week	39.4 (37.9–41.0)	36.9 (35.4–38.5)	43.7 (42.2–45.3)	41.6 (40.2–42.9)	
Hardly ever/never	15.1 (14.0–16.3)	19.9 (18.6–21.2)	15.4 (14.3–16.5)	14.8 (13.9–15.8)	
**Eating fruits**					
At least once a day	57 (55.4–58.6)	58.9 (57.4–60.5)	47.2 (45.7–48.7)	48.2 (46.9–49.6)	<0.0001
A few times a week	28 (26.6–29.4)	26.4 (25.0–27.8)	39.4 (37.9–41.0)	37.4 (36.1–38.7)	
Hardly ever/never	15 (14.0–16.2)	14.7 (13.6–15.8)	13.4 (12.4–14.5)	14.4 (13.4–15.3)	
**Driving under the influence of alcohol (last 12 months)**	20.5 (19.2–21.9)	17.7 (16.4–19.1)	10.1 (9.3–11.0)	5.5 (5.0–6.2)	<0.0001
**Driving under the influence of drugs (last 12 months)**	11.5 (10.5–12.6)	9.4 (8.4–10.5)	7.9 (7.2–8.7)	5.1 (4.6–5.7)	<0.0001

**Table 3:** Trends of the prevalence of health risk behaviors and health conditions by sex (survey waves 2008, 2011, 2015, and 2018).

	Sex	2008	2011	2015	2018	*p*
**Lie/Bet**	Males	16.4 (14.8–18.2)	15.9 (14.5–17.6)	11.3 (10.1–12.6)	10.7 (9.7–11.8)	<0.0001
	Females	4.4 (3.6–5.3)	3.1 (2.4–4.0)	1.9 (1.4–2.6)	2.6 (2.0–3.4)	<0.0001
**Smoke**						
Regular smokers	Males	21.3 (19.3–23.6)	19.2 (17.6–20.9)	19.6 (18.0–21.2)	17.4 (16.1–18.8)	0.01
Occasional smokers		11.6 (10.2–13.2)	12.9 (11.5–14.4)	14.1 (12.8–15.6)	12.7 (11.5–13.9)	
Nonsmokers		67.1 (64.6–69.4)	67.9 (65.9–69.9)	66.3 (64.4–68.2)	69.9 (68.2–71.5)	
Regular smokers	Females	22.6 (21.0–24.2)	23.3 (21.4–25.2)	21.5 (19.8–23.3)	21.2 (19.6–22.8)	0.17
Occasional smokers		16.4 (14.9–18.0)	14.9 (13.3–16.6)	17.7 (16.1–19.5)	17.2 (15.8–18.8)	
Nonsmokers		61 (59.1–63.0)	61.8 (59.7–64.1)	60.8 (58.6–62.9)	61.6 (59.7–63.5)	
**Binge drinking at least once in the last month**	Males	38 (35.6–40.5)	37 (34.9–39.2)	35.5 (33.5–37.5)	35.5 (33.8–37.3)	0.26
	Females	26.2 (24.4–28.0)	27.4 (25.4–29.5)	27.7 (25.8–29.8)	31.1 (29.2–33.0)	0.0037
**Being drunk at least once in the last 12 months**	Males	47.5 (45.0–50.0)	49.5 (47.3–51.7)	49.3 (47.2–51.4)	47.2 (45.3–49.1)	0.33
	Females	45.9 (43.9–48.0)	45.9 (43.6–48.3)	45 (42.7–47.3)	49.3 (47.2–51.3)	0.04
**Being bullied in the last 12 months**	Males	19.9 (17.8–22.1)	17.3 (15.7–19.0)	18.5 (16.9–20.1)	18.3 (16.9–19.8)	0.24
	Females	21.5 (19.8–23.2)	22.1 (20.2–24.0)	21.8 (20.0–23.7)	28 (26.3–29.9)	<0.0001
**Being a bully in the last 12 months**	Males	25.4 (23.2–27.8)	25.3 (23.5–27.3)	22.4 (20.7–24.2)	14.5 (13.3–15.9)	<0.0001
	Females	11.5 (10.3–12.9)	12.5 (11.1–14.1)	11.5 (10.2–13.0)	9.1 (8.0–10.4)	<0.01
**BMI categories**						
Obese	Males	3.5 (2.2–5.6)	3.1 (2.4–3.9)	3.3 (2.7–4.2)	2.8 (2.2–3.5)	0.11
Overweight		16.3 (14.3–18.6)	14.8 (13.3–16.3)	16.4 (5.0–18.0)	16.2 (14.8–17.6)	
Underweight		2.8 (2.0–3.8)	1.6 (1.1–2.4)	3 (2.4–3.8)	1.7 (1.3–2.3)	
Obese	Females	1.8 (1.3–2.5)	1.3 (0.9–1.9)	3 (2.3–3.9)	1.8 (1.4–2.4)	<0.01
Overweight		8.8 (7.8–9.9)	10.2 (8.9–11.8)	8.5 (7.3–9.9)	9.1 (8.0–10.2)	
Underweight		3.5 (2.8–4.4)	3.1 (2.3–4.2)	4.2 (3.4–5.2)	4 (3.3–4.9)	
**High distress level**	Males	11.6 (10.2–13.2)	9.4 (8.2–10.7)	11.9 (10.7–13.2)	11.5 (10.4–12.7)	0.03
	Females	25.9 (24.2–27.7)	23.8 (21.9–25.7)	30.7 (28.7–32.7)	32.7 (30.8–34.6)	<0.0001
**Early sex (before 14 years old)**	Males	4.4 (3.6–5.5)	4 (3.2–4.9)	4.3 (3.5–5.2)	3.3 (2.7–4.0)	0.25
	Females	2.8 (2.2–3.6)	2.1 (1.5–2.8)	2.3 (1.7–3.0)	2.7 (2.1–3.4)	0.41
**No condom use during the last sexual intercourse**	Males	24.9 (21.9–28.1)	30.8 (27.6–34.1)	36.4 (33.4–39.5)	36.4 (33.6–39.3)	<0.0001
	Females	39.8 (36.8–42.8)	43.6 (40.3–47.0)	46.6 (43.2–50.1)	47.2 (44.0–50.5)	<0.01
**Use of cannabis in the last month**	Males	27.8 (25.8–29.9)	25.2 (23.4–27.2)	32.6 (30.8–34.5)	29 (27.3–30.6)	<0.0001
	Females	22.6 (21.0–24.3)	17.6 (16.0–19.3)	23.4 (21.6–25.3)	25 (23.3–26.8)	<0.0001
**Use of cocaine in the last month**	Males	3.6 (2.9–4.4)	3.4 (2.7–4.3)	2.6 (2.1–3.3)	1.5 (1.2–2.0)	<0.0001
	Females	2.4 (1.9–3.1)	1.5 (1.0–2.1)	1.4 (1.0–2.0)	1.4 (1.0–1.9)	0.01
**Physical activity**						
At least once a week	Males	80 (78.0–81.7)	80.7 (79.0–82.3)	81.7 (80.1–83.2)	78.6 (77.0–80.1)	0.06
Less than once a week/hardly ever/never		20 (18.3–22.0)	19.3 (17.7–21.0)	18.3 (16.8–19.9)	21.4 (19.9–23.0)	
At least once a week	Females	64.3 (62.3–66.2)	66.8 (64.7–68.9)	68.1 (66.0–70.1)	68.6 (66.7–70.4)	0.01
Less than once a week/hardly ever/never		35.7 (33.8–37.7)	33.2 (31.1–35.3)	31.9 (29.9–34.0)	31.4 (29.6–33.3)	
**Eating vegetables**						
At least once a day	Males	39.4 (36.9–42.0)	37.2 (35.1–39.4)	34.4 (32.5–36.4)	39.1 (37.3–41.0)	<0.0001
Hardly ever/never		18.3 (16.6–20.2)	24.5 (22.7–26.4)	18 (16.5–19.7)	17.2 (15.8–18.6)	
At least once a day	Females	51.9 (49.8–53.9)	49.5 (47.2–51.8)	47.7 (45.4–49.9)	48.4 (46.4–50.4)	<0.01
Hardly ever/never		11.7 (10.4–13.1)	15 (13.4–16.8)	12.6 (11.2–14.2)	12.3 (11.0–13.7)	
**Eating fruits**						
At least once a day	Males	53.1 (50.6–55.6)	56.3 (54.1–58.5)	42.9 (40.9–44.9)	46 (44.2–47.9)	<0.0001
Hardly Ever/never		15.9 (14.3–17.7)	15.3 (13.8–16.9)	14.3 (12.9–15.8)	13.8 (12.5–15.1)	
At least once a day	Females	61.1 (59.1–63.0)	61.6 (59.4–63.9)	51.7 (49.5–54.0)	50.7 (48.7–52.7)	<0.0001
Hardly ever/never		14.1 (12.8–15.6)	14.1 (12.6–15.7)	12.5 (11.1–14.0)	15 (13.6–16.5)	
**Driving under the influence of alcohol (last 12 months)**	Males	28 (25.9–30.2)	23.8 (21.8–25.9)	14.6 (13.3–16.1)	8 (7.1–9.0)	<0.0001
	Females	11.1 (9.7–12.6)	9.6 (8.2–11.2)	5.2 (4.4–6.2)	2.9 (2.3–3.7)	<0.0001
**Driving under the influence of drugs (last 12 months)**	Males	14.8 (13.3–16.5)	12.2 (10.7–13.8)	12.1 (10.8–13.5)	7.4 (6.5–8.4)	<0.0001
	Females	7.2 (6.1–8.5)	5.7 (4.6–7.0)	3.4 (2.7–4.2)	2.6 (2.1–3.3)	<0.0001

**Table 4:** Trends of the prevalence of health risk behaviors and health conditions by high–school years (survey waves 2008, 2011, 2015, and 2018).

	School Year	2008	2011	2015	2018	*p*
**Lie/Bet**	Biennium	8.8 (7.3–10.5)	5.6 (4.5–7.0)	4.3 (3.4–5.6)	4.7 (3.8–5.7)	<0.0001
	Triennium	11.7 (10.5–13.0)	12.3 (11.1–13.6)	8.4 (7.5–9.4)	8.2 (7.4–9.1)	<0.0001
**Smoke**						
Regular smokers	Biennium	11.7 (9.5–14.2)	11.1 (9.5–12.9)	11.7 (10.1–13.4)	10 (8.7–11.5)	0.14
Occasional smokers		14 (12.3–16.0)	13.8 (12.0–15.9)	16.5 (14.6–18.6)	13.4 (11.9–15.1)	
Nonsmokers		74.3 (71.5–76.9)	75.1 (72.6–77.4)	71.8 (69.4–74.1)	76.6 (74.6–78.5)	
Regular smokers	Triennium	28.5 (26.9–30.2)	27.6 (26.0–29.3)	26.5 (24.9–28.1)	25.4 (24.0–26.9)	0.04
Occasional smokers		13.9 (12.6–15.2)	13.9 (12.6–15.2)	15.4 (14.2–16.8)	15.9 (14.7–17.1)	
Nonsmokers		57.6 (55.8–59.4)	58.5 (56.7–60.4)	58.1 (56.3–59.9)	58.7 (57.1–60.3)	
**Binge drinking at least once in the last month**	Biennium	22.4 (19.9–25.4)	19.9 (17.7–22.2)	20.5 (18.4–22.8)	22.6 (20.6–24.7)	0.28
	Triennium	38.4 (36.6–40.3)	40.2 (38.3–42.1)	38.8 (37.0–40.6)	40.2 (38.6–41.9)	0.37
**Being drunk at least once in the last 12 months**	Biennium	32.5 (29.7–35.5)	29.4 (26.8–32.0)	27.8 (25.4–30.3)	31.2 (28.9–33.5)	0.06
	Triennium	55.6 (53.7–57.5)	59.1 (57.2–60.9)	59.4 (57.6–61.2)	58.9 (57.2–60.6)	0.01
**Being bullied in the last 12 months**	Biennium	24.2 (21.6–27.0)	22 (19.7–24.4)	22.4 (20.2–24.6)	25.6 (23.6–27.7)	0.11
	Triennium	18.4 (17.0–19.8)	18.1 (16.7–19.5)	18.5 (17.2–20.0)	21.3 (19.9–22.6)	<0.01
**Being a bully in the last 12 months**	Biennium	18.7 (16.2–21.5)	18.3 (16.2–20.6)	16.1 (14.3–18.1)	12.2 (10.7–13.9)	<0.0001
	Triennium	18.7 (17.3–20.2)	19.6 (18.2–21.1)	17.8 (16.5–19.2)	11.7 (10.7–12.8)	<0.0001
**BMI categories**						
Obese	Biennium	3.5 (1.9–6.3)	1.8 (1.3–2.7)	3.8 (3.0–5.0)	2 (1.5–2.7)	<0.01
Overweight		11 (8.8–13.7)	11.3 (9.6–13.1)	15 (13.1–16.9)	13 (11.6–14.7)	
Underweight		4.8 (3.7–6.2)	3.1 (2.1–4.5)	2.9 (2.1–4.0)	2.6 (1.9–3.4)	
Obese	Triennium	2.2 (1.7–2.8)	2.4 (1.9–3.1)	2.7 (2.2–3.4)	2.5 (2.1–3.1)	<0.0001
Overweight		13.7 (12.5–15.0)	13.4 (12.2–14.8)	11.1 (10.0–12.2)	12.5 (11.4–13.6)	
Underweight		2.1 (1.6–2.7)	1.9 (1.4–2.5)	4 (3.4–4.8)	3 (2.5–3.7)	
**High distress level**	Biennium	16.3 (14.4–18.3)	13.7 (11.9–15.7)	17.9 (16.0–20.0)	19.1 (17.3–21.1)	0.001
	Triennium	20 (18.6–21.5)	18.1 (16.7–19.5)	23 (21.5–24.6)	23.5 (22.1–24.9)	<0.0001
**Early sex (before 14 years old)**	Biennium	4.6 (3.6–5.9)	3.1 (2.3–4.2)	3.3 (2.4–4.4)	2.9 (2.2–3.9)	0.08
	Triennium	3 (2.5–3.7)	3 (2.5–3.8)	3.3 (2.7–4.0)	3 (2.5–3.6)	0.92
**No condom use during the last sexual intercourse**	Biennium	19.3 (14.9–24.6)	31 (25.1–37.6)	35 (29.3–41.1)	33.5 (28.2–39.1)	<0.001
	Triennium	35.6 (33.3–38.0)	38.7 (36.2–41.2)	42.7 (40.2–45.1)	43.4 (41.1–45.7)	<0.0001
**Use of cannabis in the last month**	Biennium	10.8 (9.3–12.5)	9.1 (7.7–10.8)	15.7 (13.9–17.7)	16.2 (14.5–18.0)	<0.0001
	Triennium	34.6 (32.9–36.3)	29.5 (27.8–31.3)	36.6 (34.9–38.3)	34.4 (32.8–35.9)	<0.0001
**Use of cocaine in the last month**	Biennium	1 (0.6–1.6)	1 (0.6–1.7)	1 (0.6–1.7)	0.4 (0.2–0.8)	0.13
	Triennium	4.3 (3.6–5.1)	3.4 (2.8–4.2)	2.7 (2.2–3.3)	2.2 (1.8–2.7)	<0.0001
**Physical activity**						
At least once a week	Biennium	77.9 (75.5–80.0)	79.5 (77.2–81.6)	79 (76.8–81.1)	78.7 (76.7–80.6)	0.77
Less than once a week/hardly ever/never		22.1 (20.0–24.5)	20.5 (18.4–22.8)	21 (18.9–23.2)	21.3 (19.4–23.3)	
At least once a week	Triennium	68.8 (67.1.70.5)	70.4 (68.7–72.1)	72.5 (70.8–74.1)	70.4 (68.9–71.9)	0.02
Less than once a week/hardly ever/never		31.2 (29.5–32.9)	29.6 (27.9–31.3)	27.5 (26.0–29.2)	29.6 (28.1–31.1)	
**Eating vegetables**						
At least once a day	Biennium	47 (44.0–50.0)	43.6 (40.8–46.4)	39.7 (37.1–42.3)	41.3 (39.0–43.6)	<0.0001
Hardly ever/never		16 (14.2–18.2)	20.4 (18.2–22.7)	17.1 (15.4–18.9)	17.1 (15.4–18.9)	
At least once a day	Triennium	44.5 (42.7–46.4)	42.9 (41.1–44.8)	41.7 (39.9–43.5)	45.2 (43.5–46.8)	<0.0001
Hardly ever/never		14.5 (13.2–15.9)	19.6 (18.1–21.1)	14.2 (13.0–15.5)	13.3 (12.2–14.5)	
**Eating fruits**						
At least once a day	Biennium	58.6 (55.8–61.5)	62.1 (59.4–64.8)	48.3 (45.7–50.9)	48.3 (46.0–50.7)	<0.0001
Hardly Ever/never		14 (12.2–16.0)	13.3 (11.5–15.3)	12.8 (11.1–14.7)	13.1 (11.6–14.7)	
At least once a day	Triennium	55.9 (54.1–57.7)	56.9 (55.0–58.7)	46.4 (44.6–48.2)	48.3 (46.6–49.9)	<0.0001
Hardly ever/never		15.7 (14.4–17.2)	15.6 (14.3–17.0)	13.8 (12.6–15.1)	15.2 (14.1–16.5)	
**Driving under the influence of alcohol (last 12 months)**	Biennium	10.9 (9.0–13.1)	8 (6.4–9.9)	4.1 (3.2–5.3)	2 (1.4–2.8)	<0.0001
	Triennium	25.7 (24.0–27.5)	22.8 (21.1–24.6)	14.2 (13.0–15.4)	7.9 (7.0–8.8)	<0.0001
**Driving under the influence of drugs (last 12 months)**	Biennium	4.5 (3.4–5.9)	3.2 (2.3–4.6)	3.9 (3.0–5.0)	1.8 (1.4–2.5)	<0.001
	Triennium	15.3 (13.9–16.7)	12.6 (11.3–14.1)	10.6 (9.5–11.8)	7.3 (6.5–8.2)	<0.0001

**Table 5:** Trends of the prevalence of health risk behaviors and health conditions by socioeconomic status (survey waves 2008, 2011, 2015, and 2018).

	**Socioeconomic Status**	**2008**	**2011**	**2015**	**2018**	***p***
**Lie/Bet**	Low	11.2 (9.3–13.4)	10.2 (8.5–12.3)	7.9 (6.3–9.9)	6.7 (5.4–8.4)	<0.01
	Medium	10.2 (8.8–11.7)	9.4 (8.2–10.8)	6.6 (5.6–7.7)	7.5 (6.5–8.6)	<0.0001
	High	11.2 (9.4–13.3)	9.9 (8.3–11.7)	7.0 (5.8–8.6)	5.8 (4.8–7.0)	<0.0001
**Smoke**						
Regular smokers	Low	24.9 (21.7–28.4)	21.4 (18.8–24.1)	24.0 (21.3–27.0)	24.3 (21.8–27.0)	0.71
Occasional smokers		13.4 (11.4–15.7)	14.5 (12.3–17.1)	13.8 (11.7–16.3)	14.0 (11.9–16.5)	
Nonsmokers		61.7 (58.3–65.1)	64.1 (60.9–67.2)	62.2 (58.9–65.3)	61.6 (58.5–64.7)	
Regular smokers	Medium	21.3 (19.6–23.2)	22.3 (20.5–24.3)	20.6 (18.9–22.4)	19.1 (17.7–20.7)	0.05
Occasional smokers		14.0 (12.4–15.6)	13.0 (11.6–14.7)	15.5 (13.9–17.3)	15.8 (14.4–17.3)	
Nonsmokers		64.7 (62.5–66.9)	64.6 (62.4–66.8)	63.9 (61.7–66.0)	65.1 (63.2–66.9)	
Regular smokers	High	21.2 (18.8–23.7)	20.4 (18.2–22.8)	19.3 (17.2–21.5)	17.2 (15.5–19.1)	0.02
Occasional smokers		14.9 (12.9–17.1)	14.3 (12.3–16.5)	18.1 (16.0–20.4)	15.3 (13.6–17.2)	
Nonsmokers		63.9 (61.0–66.8)	65.3 (62.5–68.1)	62.6 (59.9–65.3)	67.5 (65.1–69.7)	
**Binge drinking at least once in the last month**	Low	33.5 (30.0–37.2)	32.8 (29.7–36.0)	31.9 (28.8–35.1)	36.7 (33.6–40.0)	0.23
	Medium	32.1 (29.9–34.3)	32.3 (30.1–34.6)	31.5 (29.4–33.6)	33.1 (31.2–35.0)	0.76
	High	32.0 (29.2–34.9)	33.7 (30.9–36.6)	34.4 (31.7–37.1)	32.3 (30.0–34.7)	0.56
**Being drunk at least once in the last 12 months**	Low	48.6 (45.2–52.1)	47.3 (44.0–50.7)	46.6 (43.2–50.1)	51.2 (47.8–54.5)	0.30
	Medium	46.0 (43.6–48.4)	48.5 (46.1–50.9)	46.6 (44.3–48.9)	48.9 (46.8–51.0)	0.22
	High	47.0 (43.9–50.1)	49.0 (45.9–52.0)	51.2 (48.4–54.1)	46.9 (44.4–49.5)	0.12
**Being Bullied in the last 12 months**	Low	21.1 (18.6–23.8)	21.2 (18.6–24.0)	20.8 (18.2–23.7)	27.0 (24.2–29.9)	<0.01
	Medium	20.3 (18.1–22.6)	17.7 (16.0–19.6)	18.1 (16.4–19.9)	21.3 (19.6–23.0)	0.02
	High	19.9 (17.6–22.4)	21.4 (19.0–24.0)	21.6 (19.4–24.1)	22.9 (20.9–25.1)	0.3735
**Being a bully in the last 12 months**	Low	21.1 (17.8–24.8)	20.6 (18.0–23.4)	17.9 (15.4–20.6)	13.3 (11.3–15.6)	<0.001
	Medium	18.1 (16.4–20.0)	16.9 (15.3–18.7)	16.3 (14.7–17.9)	10.7 (9.5–12.1)	<0.0001
	High	17.3 (15.1–19.8)	20.2 (17.9–22.7)	18.0 (16.0–20.3)	12.2 (10.7–14.0)	<0.0001
**BMI categories**						
Obese	Low	4.4 (3.2–6.0)	3.3 (2.3–4.8)	4.0 (2.8–5.5)	3.7 (2.7–5.1)	0.10
Overweight		17.4 (14.2–21.2)	15.2 (13.0–17.8)	14.7 (12.5–17.3)	14.7 (12.6–17.1)	
Underweight		3.1 (2.1–4.4)	1.5 (0.9–2.7)	4.1 (2.9–5.7)	2.7 (1.8–4.1)	
Obese	Medium	2.3 (1.1–4.9)	2.2 (1.6–3.1)	2.7 (2.0–3.5)	2.1 (1.6–2.8)	0.88
Overweight		12.2 (10.7–13.7)	12.6 (11.1–14.3)	12.6 (11.2–14.2)	12.9 (11.7–14.3)	
Underweight		2.5 (1.8–3.4)	2.2 (1.5–3.1)	2.9 (2.0–3.5)	2.9 (2.3–3.7)	
Obese	High	1.5 (0.8–2.5)	1.4 (0.8–2.3)	2.0 (1.4–2.9)	1.7 (1.2–2.4)	0.17
Overweight		9.0 (7.4–10.9)	10.9 (9.2–12.8)	11.6 (9.9–13.5)	11.8 (10.2–13.5)	
Underweight		4.2 (3.1–5.8)	3.0 (2.0–4.6)	4.1 (3.1–5.3)	2.6 (1.9–3.5)	
**High distress level**	Low	20.6 (18.3–23.2)	18.9 (16.6–21.6)	22.3 (19.6–25.1)	25.6 (22.9–28.5)	<0.01
	Medium	17.9 (16.2–19.6)	15.2 (13.7–16.9)	20.5 (18.7–22.3)	21.6 (20.0–23.3)	<0.0001
	High	18.0 (15.8–20.4)	16.2 (14.2–18.5)	23.0 (20.7–25.4)	19.7 (17.8–21.7)	<0.001
**Early sex (before 14 years old)**	Low	3.5 (2.5–4.8)	2.7 (1.8–4.0)	3.2 (2.2–4.6)	3.7 (2.7–5.0)	0.64
	Medium	3.3 (2.6–4.3)	2.6 (2.0–3.4)	2.4 (1.8–3.2)	2.3 (1.8–2.9)	0.15
	High	4.0 (2.9–5.4)	3.9 (2.9–5.3)	4.8 (3.7–6.2)	3.3 (2.5–4.4)	0.37
**No condom use during last sexual intercourse**	Low	35.7 (31.0–40.6)	40.9 (36.2–45.8)	45.2 (40.3–50.2)	46.3 (41.7–51.1)	<0.01
	Medium	32.1 (29.1–35.3)	35.6 (32.3–39.0)	40.3 (37.0–43.7)	39.1 (36.1–42.3)	<0.01
	High	29.3 (25.2–33.8)	38.1 (33.7–42.8)	39.8 (35.6–44.2)	43.3 (39.3–47.4)	<0.001
**Use of cannabis in the last month**	Low	24.0 (21.6–26.7)	21.1 (18.5–23.8)	29.4 (26.5–32.4)	29.6 (26.8–32.6)	<0.0001
	Medium	25.3 (23.4–27.2)	21.6 (19.7–23.5)	25.8 (24.0–27.8)	27.1 (25.4–28.9)	<0.001
	High	27.5 (25.0–30.3)	23.4 (21.3–26.2)	33.3 (30.7–35.9)	27.4 (25.2–29.6)	<0.0001
**Use of cocaine in the last month**	Low	2.8 (2.0–3.9)	2.3 (1.5–3.4)	2.0 (1.3–3.2)	1.3 (0.8–2.1)	0.09
	Medium	3.2 (2.5–4.0)	2.3 (1.7–3.1)	1.6 (1.2–2.2)	1.3 (0.9–1.8)	<0.0001
	High	3.0 (2.1–4.1)	2.5 (1.8–3.6)	2.8 (2.0–3.7)	1.7 (1.2–2.4)	0.14
**Physical Activity**						
At least once a week	Low	64.6 (61.5–67.6)	64.2 (60.9–67.3)	64.3 (61.0–67.5)	63.0 (59.8–66.1)	0.96
Less than once a week/hardly ever/never		35.4 (32.4–38.5)	35.8 (32.7–39.1)	35.7 (32.5–39.0)	37.0 (33.9–40.2)	
At least once a week	Medium	74.8 (72.8–76.7)	75.2 (73.2–77.1)	77.1 (75.1–78.9)	75.5 (73.8–77.2)	0.35
Less than once a week/hardly ever/never		25.2 (23.3–27.2)	24.8 (22.9–26.9)	22.9 (21.1–24.9)	24.5 (22.8–26.2)	
At least once a week	High	76.3 (73.6–78.8)	80.0 (77.6–82.2)	80.5 (78.2–82.5)	79.2 (77.1–81.2)	0.06
Less than once a week/hardly ever/never		23.7 (21.2–26.4)	20.0 (17.8–22.4)	19.5 (17.5–21.8)	20.8 (18.8–22.9)	
**Eating Vegetables**						
At least once a day	Low	41.4 (38.0–45.0)	37.4 (34.2–40.7)	31.9 (28.8–35.1)	37.1 (34.1–40.2)	<0.0001
Hardly ever/never		15.7 (13.5–18.1)	23.0 (20.4–25.8)	20.6 (18.0–23.5)	17.9 (15.6–20.5)	
At least once a day	Medium	46.2 (43.8–48.6)	43.1 (40.8–45.5)	41.6 (39.4–43.8)	42.6 (40.6–44.6)	<0.0001
Hardly ever/never		15.1 (13.5–16.9)	19.7 (17.9–21.7)	15.9 (14.3–17.6)	14.4 (13.1–15.9)	
At least once a day	High	49.5 (46.5–52.6)	47.5 (44.6–50.5)	46.5 (43.7–49.3)	50.3 (47.9–52.8)	<0.0001
Hardly ever/never		13.4 (11.4–15.7)	17.1 (14.9–19.4)	11.2 (9.6–13.1)	12.0 (10.4–13.7)	
**Eating Fruits**						
At least once a day	Low	51.3 (47.9–54.7)	49.5 (46.2–52.9)	39.7 (36.4–43.0)	45.3 (42.1–48.5)	<0.0001
Hardly ever/never		19.0 (16.7–21.6)	20.7 (18.2–23.5)	14.9 (12.8–17.5)	16.4 (14.2–18.9)	
At least once a day	Medium	58.5 (56.2–60.9)	60.5 (58.2–62.8)	47.9 (45.6–50.1)	46.7 (44.7–48.8)	<0.0001
Hardly ever/never		14.6 (13.0–16.3)	13.2 (11.7–14.8)	14.3 (12.8–15.9)	14.8 (13.4–16.2)	
At least once a day	High	60.7 (57.7–63.6)	63.4 (60.4–66.2)	51.0 (48.2–53.8)	53.1 (50.7–55.6)	<0.0001
Hardly ever/never		11.3 (9.5–13.4)	12.8 (11.0–14.9)	10.5 (8.8–12.3)	11.5 (10.0–13.2)	
**Driving under the influence of alcohol (last 12 months)**	Low	20.3 (17.8–23.1)	14.5 (12.2–17.2)	9.4 (7.7–11.5)	6.1 (4.7–7.8)	<0.0001
	Medium	20.9 (18.9–22.9)	17.8 (15.9–19.9)	9.8 (8.7–11.1)	5.2 (4.4–6.2)	<0.0001
	High	20.9 (18.3–23.8)	20.4 (17.8–23.2)	12.3 (10.6–14.1)	6.2 (5.1–7.5)	<0.0001
**Driving under the influence of drugs (last 12 months)**	Low	9.4 (7.7–11.4)	9.4 (7.5–11.8)	8.9 (7.2–10.9)	5.4 (4.1–6.9)	<0.01
	Medium	12.1 (10.6–13.8)	9.4 (8.0–11.1)	7.5 (6.5–8.7)	4.9 (4.1–5.8)	<0.0001
	High	12.8 (10.7–15.2)	10.0 (8.1–12.1)	8.6 (7.2–10.2)	5.6 (4.6–6.8)	<0.0001

## Data Availability

The data that support the findings of this study are available from the corresponding author, V.L., upon reasonable request.
